# Impact of the COVID-19 Pandemic on the Psychological Distress of Medical Students in Japan: Cross-sectional Survey Study

**DOI:** 10.2196/25232

**Published:** 2021-02-18

**Authors:** Yoshito Nishimura, Kanako Ochi, Kazuki Tokumasu, Mikako Obika, Hideharu Hagiya, Hitomi Kataoka, Fumio Otsuka

**Affiliations:** 1 Department of General Medicine Okayama University Hospital Okayama Japan

**Keywords:** COVID-19, online education, depression, pandemic, anxiety, medical student

## Abstract

**Background:**

The COVID-19 pandemic has negatively affected medical education. However, little data are available about medical students’ distress during the pandemic.

**Objective:**

This study aimed to provide details on how medical students have been affected by the pandemic.

**Methods:**

A cross-sectional study was conducted. A total of 717 medical students participated in the web-based survey. The survey included questions about how the participants’ mental status had changed from before to after the Japanese nationwide state of emergency (SOE).

**Results:**

Out of 717 medical students, 473 (66.0%) participated in the study. In total, 29.8% (141/473) of the students reported concerns about the shift toward online education, mostly because they thought online education would be ineffective compared with in-person learning. The participants’ subjective mental health status significantly worsened after the SOE was lifted (*P*<.001). Those who had concerns about a shift toward online education had higher odds of having generalized anxiety and being depressed (odds ratio [OR] 1.97, 95% CI 1.19-3.28) as did those who said they would request food aid (OR 1.99, 95% CI 1.16-3.44) and mental health care resources (OR 3.56, 95% CI 2.07-6.15).

**Conclusions:**

Given our findings, the sudden shift to online education might have overwhelmed medical students. Thus, we recommend that educators inform learners that online learning is not inferior to in-person learning, which could attenuate potential depression and anxiety.

## Introduction

The COVID-19 global pandemic has drastically changed our lives, with more than 93 million cases and 2 million deaths reported globally as of January 17, 2021, according to statistics from the World Health Organization [1]. The pandemic led to significant declines in the global economy, and the uncertainty and fears associated with COVID-19 led to increases in mental health disorders. According to a systematic review by Xiong and colleagues, high rates of anxiety, depression, posttraumatic stress disorder, and psychological distress were reported in the general population [2]. Among the people affected, health care workers (HCWs) are considered to be more vulnerable to these issues. A previous cross-sectional study performed in the Asian-Pacific region looked into the psychological impact of COVID-19 on HCWs and noted that the burden of COVID-19 cases might not be related to psychological adversity; however, the increasing number of cases and mortality of COVID-19 continues to threaten the well-being of HCWs [3]. In Japan, more than 300,000 cases have been confirmed as of January 17, 2021, with an explosion in the number of cases in early April, August, and November 2020 [4]. While Japan previously brought the outbreak under control through active cluster tracing, restriction of mass gatherings, and advocating universal masking and hand hygiene, the country has been experiencing a surge in the number of cases since November 2020, which suggests there were no simple solutions to this issue [5]. After the surge in COVID-19 cases, on April 16, 2020, the Government of Japan declared a state of emergency (SOE) in all 47 prefectures, which lasted until May 25, 2020 [6]. In Okayama, a prefecture in the western part of mainland Japan [7] with approximately 1.9 million people and ranked 20 out of 47 by population among the 47 prefectures, only 2071 confirmed cases had been reported by January 17, 2021. Behind the scenes of successful COVID-19 mitigation, however, those in education-related jobs and medical students struggled with the rapid change in the educational system, as shown in examples from Australia and Spain [8,9].

The SOE was lifted on May 14, 2020, in Okayama based on the low incidence of COVID-19. Okayama University School of Medicine (OUSM), one of the largest national universities in Japan, requested its students to stay home to prevent the spread of COVID-19 within the medical school and the hospital even before the SOE (ie, March 25, 2020). Until the stay-home request was lifted on May 22, 2020, medical students were required to cope with this sudden change in their lifestyle and shifted to online education amid the fear and considerable uncertainty surrounding COVID-19. In the early 2000s, the SARS-CoV outbreak had a devastating impact on academic education, including sudden curriculum changes and rapid integration of information technology [10-12]. Similarly, the current COVID-19 pandemic has provoked significant turmoil in society. In particular, as briefly noted above, mental health problems due to the pandemic have drawn attention worldwide as studies have suggested the need for mental health care interventions during the outbreak [13-18]. Medical students, who essentially need clinical exposure, may have been impacted even further by the pandemic because of the cancellation of clinical rotation. Due to the pandemic that forced educational institutions to eliminate in-person teaching sessions, medical students needed to adapt to new educational environments, such as distance or remote e-learning [19]. While some researchers argued that the COVID-19 pandemic could be an opportunity to catalyze changes in medical education [20], the rapid change in the system and environment might cause significant stress to medical students. Even before the COVID-19 pandemic, the global prevalence of anxiety among medical students was estimated to be 33.8%, and it was the most prevalent in those from the Middle East and Asia [21]. Also, a meta-analysis done in 2016 showed that depression was prevalent in 28.0% of medical students globally. Given the significant psychological distress related to the pandemic [22], the current prevalence of anxiety and depression among medical students might be even higher.

In Japan, medical schools follow an undergraduate medical education system, which is similar to most countries. Students typically enter medical school immediately after high school graduation, often at 18 years old, and they go through 6 years of medical education before graduation. The fundamental philosophy of academic medicine is to provide a quality educational experience. To date, few published studies have investigated the living environment and mental health status of medical students during the COVID-19 pandemic [23-25]. To address this, we conducted a total population survey of OUSM medical students to comprehensively clarify what they require and how they have been mentally affected by COVID-19.

## Methods

### Study Design, Setting, and Participants

We performed a cross-sectional study that employed an anonymous, self-administered, voluntary web-based survey. Participants included medical students in all years of study at OUSM. We invited all 717 medical students who belonged to the OUSM as of April 1, 2020 (ie, the first day of the academic year in Japan), to participate in the survey. The participants’ consent was implied by the completion of the survey.

Our research team developed the survey through consultation with a medical education expert panel at OUSM and through piloting. We conducted a pretest of all the instruments in a sample group of 15 recent medical school graduates, who graduated in April 2020, to confirm their comprehension of the test and to make sure the questions were appropriate to measure psychological distress amid the pandemic. Cronbach α for the instrument developed by the team was .73. Content validity of the survey, as confirmed by the medical education expert panel, showed that the instrument represented the proposed domains (ie, depression and anxiety distress) to be measured in medical students. The survey was administered with Qualtrics (Qualtrics International Inc), a web-based survey platform. We provided survey instructions and instruments in Japanese. We distributed survey links to the students using OUSM official mailing lists. All participants were invited to complete the survey within 1 week (ie, June 8-14, 2020, in Japan Standard Time). No financial incentives were provided for their participation in the survey. The survey included entries on demographics (ie, age, gender, education before entering medical school, employment status on the date of response, changes in employment status due to the COVID-19 pandemic, marital status, living environment, household size, and comorbidities) as well as COVID-19-related items (eg, chance of contracting COVID-19 during the current pandemic), self-learning-associated activities (eg, average amount of time spent self-learning per day), validated depression and anxiety scale instruments, and financial situations. Self-learning is defined as “proactive processes that students use to acquire academic skill” [26]. In this study, the concept of self-learning by participants includes reading medical books, watching webinars, or preparing for shelf exams by themselves. To protect participants’ anonymity as much as possible, participants were not prompted to enter their year of study.

### Measurements

#### COVID-19-Related Questions

To evaluate the extent of participants’ concerns and preparedness for COVID-19, they were asked the questions listed in Textbox 1.

**Survey questions related to COVID-19.**

COVID-19-related questions:

- What do you think the chances are that you will contract COVID-19 during the current pandemic?
- What is your degree of concern about the health status of your family?
- Do you think you have enough information about the symptoms of COVID-19?
- Do you think you have enough information about prevention and treatment of COVID-19?
- Do you feel worried about COVID-19?

To further assess students’ concerns, participants were prompted to respond to the following statements:

- I am concerned because my future career formation may be negatively affected due to the COVID-19 pandemic.
- I am concerned because the COVID-19 pandemic may attenuate our relationship to teachers.
- I am concerned because of the disruption to ongoing research or extracurricular activities.
- I am concerned about the shift toward online education.

All questions were evaluated on a 5-point Likert scale, except for the item on the health status of the family, which was evaluated on a 3-point Likert scale. Those who responded with *very concerned* or *concerned* regarding the entry on concern about the shift toward online education were prompted to provide reasons. To describe participants’ needs, they were asked to note the types of support they wish to receive from the university if there is a resurgence of COVID-19. These responses were mandatory.

#### Self-Learning and Related Activities

As noted above, we developed the survey through consultation with a medical education expert panel at our facility. We assumed that several factors might be related to changes in medical students’ subjective mental health status. To evaluate subjective mental health status and the average amount of time per day that participants stayed at home, read books, played video games, and learned by themselves, respondents were prompted to answer the following questions, as pertaining to before the SOE order (ie, April 16, 2020) and during the last 2 weeks, with the base date of when participants completed the survey: How many hours a day did you stay at home? How many hours a day did you read books? How many hours a day did you play video games? and How many hours a day did you self-learn?

#### Depression and Anxiety Disorders

We assessed the presence of depression using the 9-item Patient Health Questionnaire (PHQ-9), a common screening tool for mood disorders. We used the validated Japanese translation of the scale [27]. The total score for the PHQ-9 ranges from 0 to 27, and we defined scores of 10 or more as having *depression*. We screened for anxiety disorders using the Japanese version of the 7-item Generalized Anxiety Disorder scale (GAD-7), which was validated in 2010 [28]. The total score ranges from 0 to 21, and we defined scores of 10 or more as having *anxiety*. We used cut points of 10 or greater in this study because total scores of 10 or more in the PHQ-9 or GAD-7 represent moderate depression or anxiety, respectively. Both instruments ask respondents about their mental health status during the last 2 weeks.

#### Financial Situation

In Japan, university students typically live on a monthly allowance from parents. According to the latest statistics by the National Federation of University Co-operative Associations, students receive 72,810 Japanese yen (JPY) (approximately US $680) a month on average [29]. Participants were prompted to give their monthly allowance from the following options: *None*, *<30,000 JPY* (US $280), *30,000-49,999 JPY* (US $280-$467), *50,000-69,999 JPY* (US $467-$654), *70,000-99,999 JPY* (US $654-$935), and *≥100,000 JPY* (≥US $935). Respondents were also asked to answer whether they were receiving a scholarship or student loan.

### Statistical Analysis

We analyzed the data using JMP statistical software, version 13.1.0 (SAS Institute Inc). We used the Wilcoxon signed-rank test to examine differences in the amount of time participants spent on self-learning-related activities based on nonnormal distribution. For associations between categorical variables containing small sample sizes, we employed the Fisher exact test. To examine the predictive factors of categorical dependent variables, we used univariate logistic regression analyses. The threshold for significance was defined as *P*<.05.

### Ethical Approval

This study protocol was approved by the Institutional Review Board of Okayama University Hospital (reference No. 2006-029; approved on June 5, 2020).

### Data Availability Statement

The data sets generated and analyzed during this study are available from the corresponding author on reasonable request.

## Results

### Overview

The response rate to the survey was 66.0%, as 473 out of 717 OUSM students in all 6 years combined completed the survey. Participants’ demographic characteristics are summarized in Table 1. Of note, out of 473 respondents, 250 (52.9%) reported that they were engaged in part-time work, while 44 (9.3%) reported having resigned or lost their jobs due to the COVID-19 pandemic. Out of 473 respondents, 8 (1.7%) and 6 (1.3%) noted that they had a past medical history of anxiety disorders and depression, respectively.

**Table 1:** Demographic characteristics of the study participants.

Characteristic		Value (N=473)
Age in years (n=471)^a^, mean (SD), 95% CI		22.0 (3.3), 21.7-22.3
**Gender, n (%)**		
	Female	161 (34.0)
	Male	311 (65.8)
	Nonconforming	1 (0.2)
**Education before medical school, n (%)**		
	High school	434 (91.8)
	Career college	1 (0.2)
	4-year university	31 (6.6)
	Master’s degree	7 (1.5)
**Employment status, n (%)**		
	Part-time job	250 (52.9)
	Schoolwork only: no extra work	221 (46.7)
	Self-owned business	2 (0.4)
	Resigned or fired due to the pandemic	44 (9.3)
**Marital status, n (%)**		
	Single	467 (98.7)
	Married	5 (1.1)
	Missing data	1 (0.2)
**Living environment, n (%)**		
	Alone	308 (65.1)
	With family	127 (26.8)
	With a partner	20 (4.2)
	Alone: family or relatives nearby	18 (3.8)
Household size, mean (SD), 95% CI		1.7 (1.3), 1.6-1.8
**Comorbidity, n (%)**		
	None	401 (84.8)
	Asthma	37 (7.8)
	Anxiety disorder	8 (1.7)
	Depression	6 (1.3)
	Other	34 (7.2)

^a^Of the 473 respondents, 2 did not specify their age.

### COVID-19-Related Survey Items

**Figure 1:**
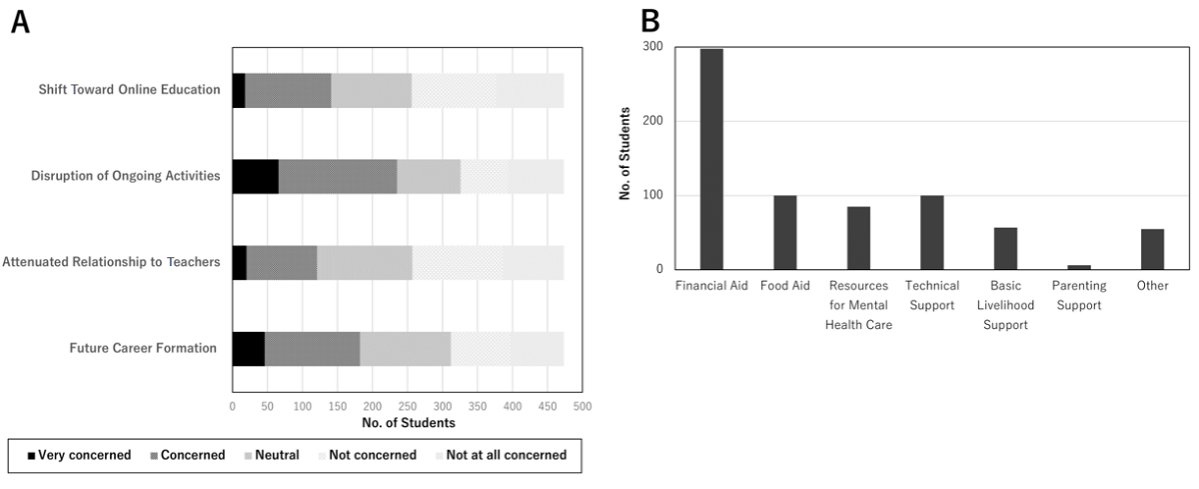
(A) The breakdown of medical students' educational concerns related to COVID-19. (B) What medical students want from the university upon the second wave the COVID-19 pandemic.

Table 2 and Figure 1 show respondents’ answers to the COVID-19-related survey items. Out of 473 respondents, 81 (17.1%) responded that they were either *likely* or *very likely* to contract COVID-19 during the ongoing pandemic; 275 (58.1%) *agreed* or *strongly agreed* to the prompt “I feel worried about COVID-19.” Regarding the breakdown of students’ concerns about COVID-19, 182 (38.5%), 121 (25.6%), and 235 (49.7%) out of 473 respondents acknowledged that they were concerned about the negative impacts of COVID-19 on their future career formation, relationship with teachers, and ongoing research or extracurricular activities, respectively, while 141 (29.8%) also reported concerns about a shift toward online education. The reasons for these concerns included the belief that online education may not be as effective as on-site education (92/141, 65.2%), possible resurgence of the COVID-19 outbreak leading to a sudden change in the curriculum (74/141, 52.5%), and decreased clinical exposure (92/141, 65.2%).

Of the 473 participants, 298 (63.0%) answered that they would request financial aid if a stay-home order recurred due to a COVID-19 resurgence, followed by a request for food aid (100/473, 21.1%), technical support for online education (100/473, 21.1%), and mental health care resources, including counseling by therapists or psychologists (85/473, 18.0%).

**Table 2:** Results of the COVID-19-related survey items.

Survey entry and responses			Value (N=473), n (%)
“**Chance of contracting COVID-19 during the current pandemic”**			
	Very likely		15 (3.2)
	Likely		66 (14.0)
	Neutral		238 (50.3)
	Unlikely		121 (25.6)
	Very unlikely		33 (6.9)
“**Degree of concern about the health status of your family”**			
	Very concerned		198 (41.9)
	Somewhat concerned		237 (50.1)
	Not concerned		38 (8.0)
“**I have enough knowledge about symptoms of COVID-19”**			
	Strongly agree		8 (1.7)
	Agree		158 (33.4)
	Neutral		169 (35.7)
	Disagree		116 (24.5)
	Strongly disagree		22 (4.7)
“**I have enough knowledge about the treatment of COVID-19”**			
	Strongly agree		6 (1.3)
	Agree		65 (13.7)
	Neutral		138 (29.2)
	Disagree		196 (41.4)
	Strongly disagree		68 (14.4)
“**I feel worried about COVID-19”**			
	Strongly agree		46 (9.7)
	Agree		229 (48.4)
	Neutral		106 (22.4)
	Disagree		77 (16.3)
	Strongly disagree		15 (3.2)
	“**Concerned about future career formation due to COVID-19”**		
		Very concerned	46 (9.7)
		Concerned	136 (28.8)
		Neutral	130 (27.5)
		Not concerned	85 (18.0)
		Never concerned	76 (16.0)
	“**Concerned about attenuated relationship to teachers”**		
		Very concerned	20 (4.2)
		Concerned	101 (21.4)
		Neutral	136 (28.7)
		Not concerned	129 (27.3)
		Never concerned	87 (18.4)
	“**Concerned about disruption of ongoing research or extracurricular activities”**		
		Very concerned	66 (14.0)
		Concerned	169 (35.7)
		Neutral	91 (19.2)
		Not concerned	67 (14.2)
		Never concerned	80 (16.9)
	“**Concerned about shift toward online education”**		
		Very concerned	18 (3.8)
		Concerned	123 (26.0)
		Neutral	115 (24.3)
		Not concerned	121 (25.6)
		Never concerned	96 (20.3)
“**The reasons for concern** **about a shift toward online education” (n=141)**^a^			
	Online education may not be as effective as on-site education		92 (65.2)
	Fear of sudden change in the curriculum		74 (52.5)
	Less clinical exposure		92 (65.2)
“**What supports do you want from the university?”**^b^			
	Financial aid (eg, tuition waiver and no-interest student loan)		298 (63.0)
	Food aid		100 (21.1)
	Resources for mental health care		85 (18.0)
	Technical support (eg, free rental of personal computer and pocket Wi-Fi)		100 (21.1)
	Basic livelihood support (eg, resources for health maintenance or preventing domestic violence)		57 (12.1)

^a^This survey entry was explicitly answered by participants who answered *very concerned* or *concerned* in response to the item “Concerned about shift toward online education.” Multiple answers were permitted.

^b^Multiple answers were permitted.

### Change in Self-Learning-Related Amount of Time Before and After the SOE

We compared the change in the average amount of time per day that respondents spent at home, reading books, playing video games, and self-learning before the SOE and within 2 weeks prior to the survey completion, after the SOE. As shown in Table 3 and Figure S1 in Multimedia Appendix 1, the participants spent significantly longer on all the activities mentioned above after the SOE compared to before the SOE (*P*<.001). There were also significant differences in their subjective mental health status before and after the SOE, based on the Wilcoxon signed-rank test (*P*<.001).

**Table 3:** Comparison of age-weighted self-learning and related activity times of medical students before and after the national state of emergency (SOE) in Japan.

Measure		Before SOE (N=473)	Last 2 weeks^a^ (N=473)	*P* value^b^
**Activity (hours/day), mean (95% CI)**				
	Stay at home	16.1 (15.7-16.6)	18.8 (18.5-19.2)	<.001
	Reading books	0.70 (0.58-0.82)	1.1 (0.96-1.2)	<.001
	Playing video games	1.5 (1.3-1.7)	2.1 (1.9-2.3)	<.001
	Self-learning	2.6 (2.4-2.8)	4.0 (3.7-4.2)	<.001
**Subjective mental health status, n (%)**				
	Very good	85 (18.0)	39 (8.2)	<.001
	Good	171 (36.1)	58 (12.2)	<.001
	Fair	193 (40.8)	286 (60.5)	<.001
	Bad	18 (3.8)	76 (16.1)	<.001
	Very bad	6 (1.3)	14 (3.0)	<.001

^a^The base date of the *last 2 weeks* is the date when the participants answered the survey. The survey period was from June 8 to 14, 2020.

^b^*P* values were calculated with the Wilcoxon signed-rank test.

### Regression Analyses of Factors Associated With Depression and Anxiety

Of the 473 participants, 75 (15.9%) had PHQ-9 scores of 10 or more and 34 (7.2%) had GAD-7 scores of 10 or more. Table 4 presents all the results from the univariate regression analyses. The odds of being depressed were significantly higher in those who had concerns about a shift toward online education (odds ratio [OR] 1.97, 95% CI 1.19-3.28) and in those who said they would request food aid (OR 1.99, 95% CI 1.16-3.44) and mental health care resources (OR 3.56, 95% CI 2.07-6.15) from the university in the event of the resurgence of COVID-19. Regarding generalized anxiety, the odds were higher in respondents who said they would request food aid (OR 2.50, 95% CI 1.21-5.20) and mental health care resources (OR 3.16, 95% CI 1.51-6.59).

**Table 4:** Results of univariate regression analyses of factors associated with depression and generalized anxiety in Japanese medical students.

Dependent variable and independent variables			Odds ratio (95% CI)^a^		*P* value	
**Depression (n=75), PHQ-9^b^ score ≥10**						
	Female vs male		1.08 (0.58-2.01)		.81	
	Age		1.06 (0.96-1.16)		.45	
	**Concerned about the following^c^:**					
		Future career formation		1.40 (0.85-2.30)		.18
		Attenuated relationship to teachers		1.46 (0.85-2.50)		.17
		Disruption of ongoing research or extracurricular activities		0.81 (0.50-1.33)		.41
		Shift toward online education		1.97 (1.19-3.28)		.009
	**Would request the following:**					
		Financial aid		0.92 (0.55-1.53)		.74
		Food aid		1.99 (1.16-3.44)		.01
		Mental health care resources		3.56 (2.07-6.15)		<.001
**Anxiety (n=34), GAD-7^d^ score ≥10**						
	Female vs male		0.81 (0.37-1.74)		.58	
	Age		0.96 (0.85-1.09)		.57	
	**Concerned about the following^c^:**					
		Future career formation		0.75 (0.36-1.58)		.45
		Attenuated relationship to teachers		1.43 (0.68-3.03)		.35
		Disruption of ongoing research or extracurricular activities		0.53 (0.26-1.09)		.09
		Shift toward online education		1.50 (0.73-3.10)		.27
	**Would request the following:**					
		Financial aid		1.25 (0.59-2.62)		.56
		Food aid		2.50 (1.21-5.20)		.01
		Mental health care resources		3.16 (1.51-6.59)		.002

^a^Univariate regression analysis was performed for each dependent variable.

^b^PHQ-9: 9-item Patient Health Questionnaire.

^c^Participants who answered *very concerned* or *concerned* in the respective survey entries listed in Table 2 were considered the *positive concerns group*. On the other hand, those who answered *neutral*, *not concerned*, or *not at all concerned* were considered the *no concerns group* in this analysis.

^d^GAD-7: 7-item Generalized Anxiety Disorder scale.

### Prevalence of Financial Hardship

Table 5 and Figure S2 in Multimedia Appendix 1 show the self-reported monthly allowance from parents and situations regarding financial aid. Out of 473 respondents, 332 (70.2%) had a monthly allowance that was lower than the national average (ie, 72,810 JPY; approximately US $680) [29], while 131 (27.7%) received some sort of financial aid for their living and education expenses.

**Table 5:** Description of the financial situation of Japanese medical students.

Characteristic		Value (N=473), n (%)
**Monthly allowance from parents (JPY^a^)**		
	None	127 (26.8)
	<30,000	43 (9.1)
	30,000-49,999	81 (17.1)
	50,000-69,999	81 (17.1)
	70,000-99,999	78 (16.5)
	≥100,000	63 (13.3)
**Financial aid**		
	None	342 (72.3)
	Student loan only	74 (15.6)
	Scholarship only	44 (9.3)
	Both student loan and scholarship	13 (2.8)

^a^JPY: Japanese yen; according to the Bank of Japan, the exchange rate of 1 US dollar to JPY was 107.39-107.41 on the morning of June 17, 2020.

## Discussion

To the best of our knowledge, this study is the first survey of Japanese medical students regarding their life circumstances and challenges due to COVID-19. As of January 17, 2021, Japan has had 307,696 cumulative COVID-19 cases, the second largest number in the World Health Organization Western Pacific region and the 39^th^ largest worldwide [1]. While Okayama has had comparatively fewer cases than other Japanese prefectures, the study results underscore that the pandemic inflicted profound mental health challenges on medical students. We found that approximately 10% of the students with part-time jobs had lost their jobs due to the pandemic. Furthermore, many medical students had concerns regarding their basic life security, demanding support from the university in the form of financial aid, food aid, technical support, and mental health care resources due to the SOE. As shown in previous studies, there may be an increasing prevalence of food insecurity during the pandemic, which might negatively affect the students’ mental well-being [30,31]. Regarding students’ subjective psychological distress, those who expressed concerns about the rapid shift toward online education and fear around basic life security were more likely to be depressed and anxious after the SOE.

Concerns around future career disruption, attenuated relationships with medical teachers, and disruption of ongoing extracurricular activities were prevalent among the participants, which underlines the considerable uncertainty amid the COVID-19 pandemic (see Table 2). In particular, 63.0% of respondents reported the need for financial aid in the event of a second wave of the pandemic. These data correspond to the fact that more than 70% of participants received no more than the national average monthly allowance from their parents (see Table 5). Contrary to the general notion in Japan that “medical students are financially well-off,” many may have experienced financial hardship. Recently, the theory of willpower has gathered public attention. Willpower is defined as the ability to resist short-term temptations to achieve long-term goals or the capacity to override an unwanted thought or impulse [32,33]. As previously studied, the theory is even applicable to students [34]. Financial stability is considered a prerequisite of willpower, which aids appropriate decision making [32]. Combined with previous research findings showing that financial instability could lead to worse mental health outcomes [35,36], educational institutions are expected to develop a strategy to offer financial support to those in need. Although there are no straightforward solutions to address psychological distress amid the pandemic, we encourage educational institutions to provide emergency grant funding and to lobby the national or local government to offer additional funding given the number of students in critical need.

Regarding lifestyle changes and psychological distress, after the SOE was lifted, the participants experienced significantly worse mental health and spent a significantly longer time at home, reading books, playing video games, and learning by themselves (see Table 3) than before the announcement of the SOE. Our results are consistent with prior longitudinal and cross-sectional studies that showed that the COVID-19 pandemic led to a sedentary lifestyle and psychological distress [13,16,17,37]. While longer self-study times and reading books could be the consequences of staying at home longer and might not have detrimental effects on students’ well-being, increasing time spent playing video games might be alarming. A previous study suggested that internet addiction is significantly associated with depression and anxiety [38]. As indicated by a previous study, the increase in gaming could be a coping behavior against psychological stress [39].

As for regression analyses of factors associated with depression and anxiety, in our study population, those who said they would request food aid and mental health care resources from the university upon the future resurgence of the COVID-19 outbreak had significantly higher odds of depression and anxiety. Furthermore, concerns around the shift toward online education were identified as factors associated with depression (see Table 4). Surprisingly, 65.2% of those concerned about the shift toward online education thought online education was less effective than in-person education. While previous studies have reported the utility and noninferiority of online learning compared to offline in-person learning [40,41], this study’s results revealed a potential gap in perception regarding the effectiveness of online education between medical students and educators. Educators should not assume that students know the potential benefits of online learning, and it is essential to inform learners that online learning is not inferior to in-person learning, which could attenuate potential depression and anxiety. While in-person communication has become difficult nowadays due to the fear of COVID-19, medical schools may need to reach out to students to find out who among them are suffering from underlying life insecurity and provide multilateral support, including early mental health care interventions. In particular, cognitive behavioral therapy (CBT) has drawn attention as an effective solution to help mitigate psychological distress. Ho et al discussed mental health strategies to support HCWs amid the COVID-19 pandemic, and they noted that CBT could mitigate maladaptive coping behaviors, including self-blame, by enhancing stress-managing skills [42]. In particular, internet CBT (I-CBT) using a learning management platform such as Moodle has been noted as a cost-effective and valid solution to address psychiatric symptoms [43,44]. Further studies are warranted to determine whether I-CBT may also be an effective solution for medical students’ psychological distress in Japan.

Although this study’s findings might be discouraging, medical students potentially have a role during the COVID-19 pandemic. In several countries including the United States and Vietnam, the mobilization of medical students toward a frontline COVID-19 response has been reported as a considerable help in assisting HCWs while increasing the clinical experience of the students [45,46]. While mobilization of medical students to help in the COVID-19 response needs to be voluntary, this is undoubtedly something that medical students can contribute to the community, health care system, and society. Knowing about good examples from other countries amid the pandemic might help medical students raise their hopes and overcome psychological distress. Educators are encouraged to provide supports to meet individual student needs, and the approach should not be a one-size-fits-all type. A longitudinal nationwide study to follow up with the mental health of medical students is warranted.

Several limitations of this study should be noted. First, due to the single-center, cross-sectional survey design, we may not conclude causal relationships. Second, cross-sectional research contains a limitation in terms of addressing changes over time. While we illustrated the changes in the average amount of time that students spent on self-learning-related activities before and after the SOE in Table 3, a longitudinal study design would be more appropriate to examine the differences in the study cohort. Third, we asked participants to provide their mental health status and amount of time spent on self-learning-related activities before the SOE, approximately 6 weeks before the survey implementation, both of which are subject to recall bias. Fourth, we did not check the convergent and structural validity of the instrument developed by our team. Thus, it should be noted that these scores might not represent hypothesized domains (ie, depression and anxiety). Next, the PHQ-9 and the GAD-7 are self-reported questionnaires that measure psychiatric symptoms, and no clinical diagnoses of depression or anxiety were made. It should be noted that the gold standard for establishing psychiatric diagnosis involves structured clinical interviews and functional neuroimaging [47,48]. Also, PHQ-9 and GAD-7 scores were obtained only after the SOE. Thus, they might not have changed during the period from before the SOE to after. Lastly, due to the nature of the survey topic, those interested in the COVID-19 public health emergency or in mental health may have been more likely to respond, leading to self-selection bias. Despite these limitations, a total population sampling strategy coupled with higher-than-usual response rates [49] contributed to high internal validity.

In conclusion, through this study, we have provided graphical data and evidence regarding the impacts of the COVID-19 pandemic on medical students. In circumstances of considerable uncertainty, both educators and medical students need to be flexible, patient, and resilient. The uncertainty and drastic change triggered substantial psychological distress in students, which was greater than we had assumed. Although Okayama prefecture survived the pandemic with fewer confirmed COVID-19 cases than other prefectures, medical students experienced significant adverse impacts due to the public health emergency. Educational institutions should recognize the prevalence of basic needs insecurity, such as financial difficulties and a shortage of staples, including food. We encourage educational institutions to offer emergency grant funding and lobby the government to provide additional funding at a policy level, given the number of students in critical need. As medical educators, we need to be accountable for the advantages of online education in the field of medicine to alleviate students’ psychological distress, in addition to providing multilateral support to those in need, including early mental health care interventions such as I-CBT. While we targeted medical students in a single Japanese national university, the survey results warrant further research and analysis to determine whether the observed distress was amplified by existing anxiety, depression, burnout, etc, or whether it was a wholly COVID-19-related phenomenon. We call for increased research in populations with more COVID-19 cases than in Japan to figure out the challenges that medical students from different cultures and backgrounds have been facing amid the pandemic.

## Appendix

Multimedia Appendix 1 Amount of time respondents spent on self-learning and related activities and their self-reported financial situation.

## Abbreviations

CBT: cognitive behavioral therapy
GAD-7: 7-item Generalized Anxiety Disorder scale
HCW: health care worker
I-CBT: internet cognitive behavioral therapy
JPY: Japanese yen
OR: odds ratio
OUSM: Okayama University School of Medicine
PHQ-9: 9-item Patient Health Questionnaire
SOE: state of emergency
