# Supplementary figures and images for: Impact of the COVID-19 Pandemic on the Psychological Distress of Medical Students in Japan: Cross-sectional Survey Study
Source: J Med Internet Res. 2021 Feb 18;23(2):e25232. doi: 10.2196/25232 (PMC7894621; doi:10.2196/25232)

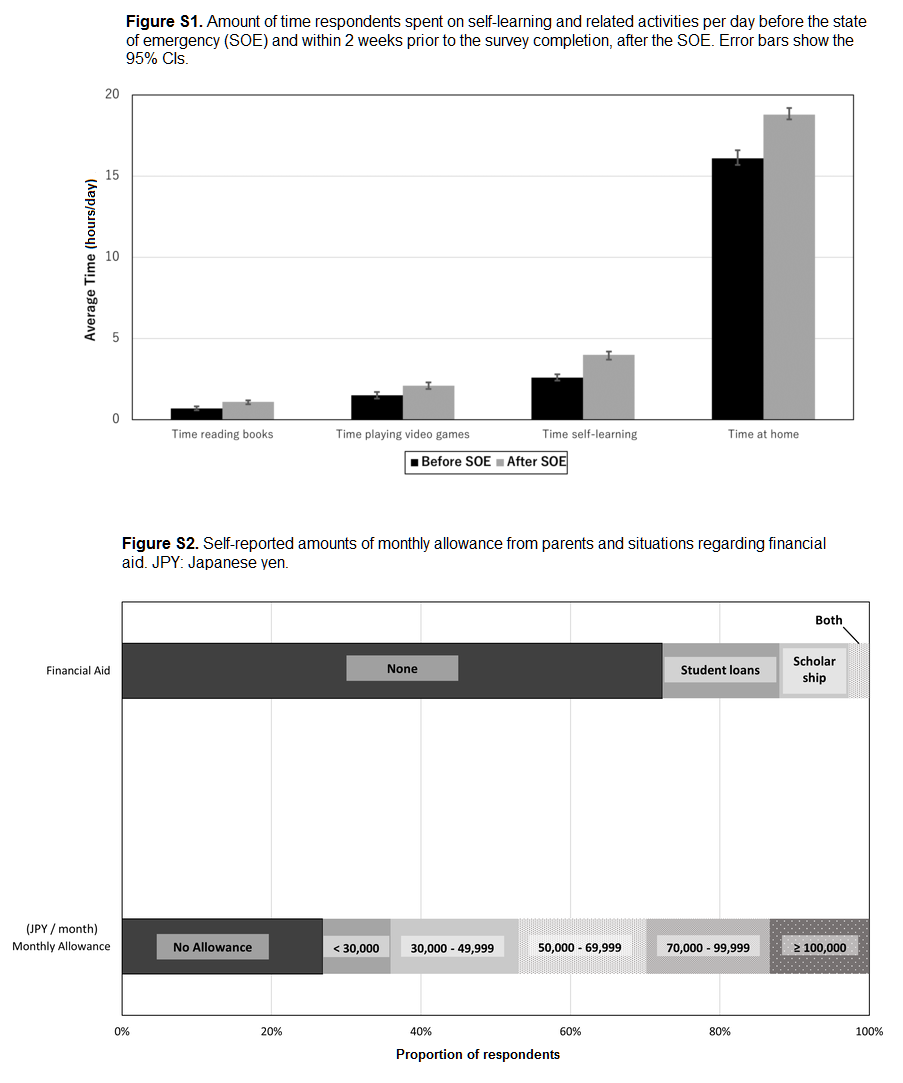

Supplement: Multimedia Appendix 1 [file jmir_v23i2e25232_app1.png]
